# Nondysraphic intramedullary spinal cord lipoma: a case report

**DOI:** 10.1097/MS9.0000000000002060

**Published:** 2024-04-17

**Authors:** Susmin Karki, Prakash Regmi, Asmita Parajuli, Khusbu Kumari, Bikas Thapa, Sushil K. Shilpakar

**Affiliations:** a Maharajgunj Medical Campus; b Department of Neurosurgery, Tribhuvan University Institute of Medicine, Maharajgunj, Nepal

**Keywords:** intramedullary, lipoma, nondysraphic, spinal cord tumor

## Abstract

**Introduction:**

Intramedullary nondysraphic spinal lipomas are extremely rare among primary spinal cord tumors. These patients present with nonspecific sensory symptoms followed by deterioration of motor symptoms. As the safety margins for neurological preservation are thin, meticulously locating the extent of the tumor and choosing the resection modalities is essential.

**Case report:**

The authors report a rare case of a 35-year-old male who presented with progressive difficulty in walking for 6 months associated with numbness and tingling sensation in the bilateral upper and lower limbs. He was diagnosed with nondysraphic intramedullary cervicothoracic lipoma and underwent subtotal resection of the tumor.

**Conclusion:**

Nondysraphic intramedullary spinal cord lipomas are rare and may present as nonspecific neurological symptoms. Hence, they should be considered differentials of intramedullary spinal cord tumors. Surgery appears to be the mainstay of treatment.

## Introduction

Highlights

- Lipoma is ubiquitous, but presenting as a nondysprahic intramedullary spinal cord lipoma in an adult is extremely rare.
- Intramedullary lipoma has a bimodal presentation, becoming symptomatic during the first years of life or the second to fifth decade.
- For better postoperative neurologic outcomes, early surgical intervention and partial or subtotal resection can be performed.

Lipoma is ubiquitous, but presenting as a nondysraphic intramedullary spinal tumor is rare^1^. The incidence rate of primary spinal cord tumors is 0.74–2.5 per 100 000 persons^2^. Although a low-grade tumor, a lipoma can cause significant neurological deficits because of its location and mass effect^3^. There is a bimodal age distribution presenting mainly with nonspecific sensory symptoms followed by motor deficits. Early tumor debulking, either by gross total, subtotal, or partial resection, can improve postoperative neurological outcomes. This case was reported as following the Surgical CAse REport (SCARE) guidelines 2023^4^.

## Case presentation

A 35-year-old male presented to the neurosurgery outpatient department with complaints of progressive difficulty in walking for 6 months associated with numbness and tingling sensation of the bilateral upper and lower limbs for 2 months. Numbness on the upper limbs gradually progressed to bilateral lower limbs over 3 months. The patient denies a history of fever, trauma, abnormal body movements, headache, drowsiness, loss of consciousness, and vomiting. He has normal bowel and bladder habits with no features of incontinence. He has no co-morbidities, takes a mixed diet, consumes alcohol daily, and has smoked 20 pack years.

The patient was conscious, cooperative, and well-oriented to time, place, and person. He had stable vitals and had no lymphadenopathy or clubbing. On external examination, signs of skin stigmata such as dimpling, hair, or mass were absent over the vertebral region. On the nervous system examination, his higher mental functions were intact, with a Glasgow coma scale of E4V5M6. Bilateral pupils were 2 mm in size, round, regular, and reactive to light. All 12 pairs of cranial nerves were intact. The Medical Research Council (MRC) scale for the power of the right elbow on flexion and extension was 5/5, whereas, on the left, it was 4/5 on flexion and 3/5 on extension. The wrist had 5/5 power bilaterally. The right-hand fingers had full power on the finger adduction test, whereas the left had only 3/5. The bulk and tone were normal bilaterally in both upper and lower limbs. Reflexes were brisk in the upper and lower limbs with upgoing plantar on the bilateral lower limb. Clonus was present on the left lower limb. The sensations were normal and equal bilaterally on all the dermatomes. The tone of the anal canal was also normal. The cerebellar function was normal. Other system examinations were within normal limits.

**Figure 1:**
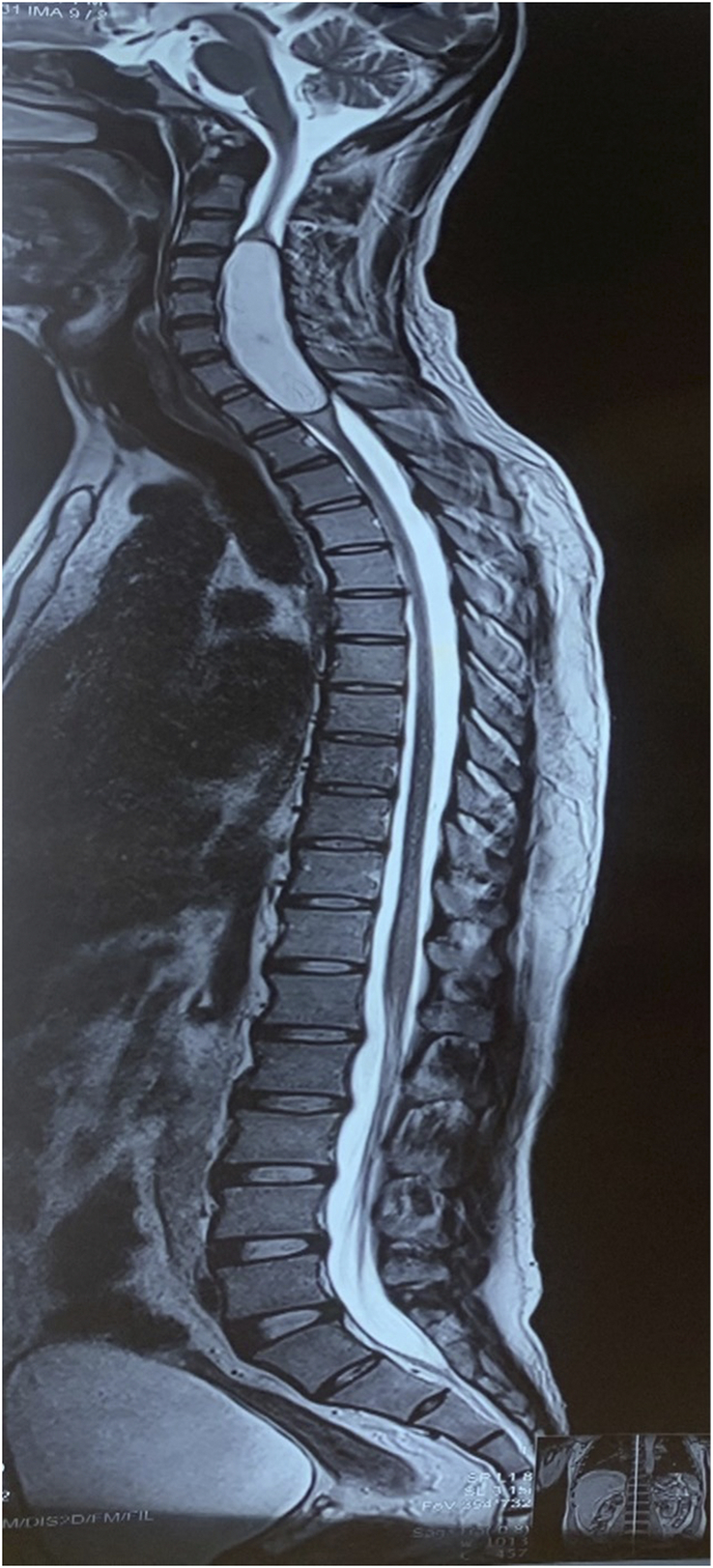
T2 weighted sagittal image of whole spine showing well defined, ~7.6×1.8×1.5 cm size high signal intensity lesion in intradural intramedullary compartment of cervical spine extending from C3 to D1 level. No feature suggestive of spinal dysraphism.

**Figure 2:**
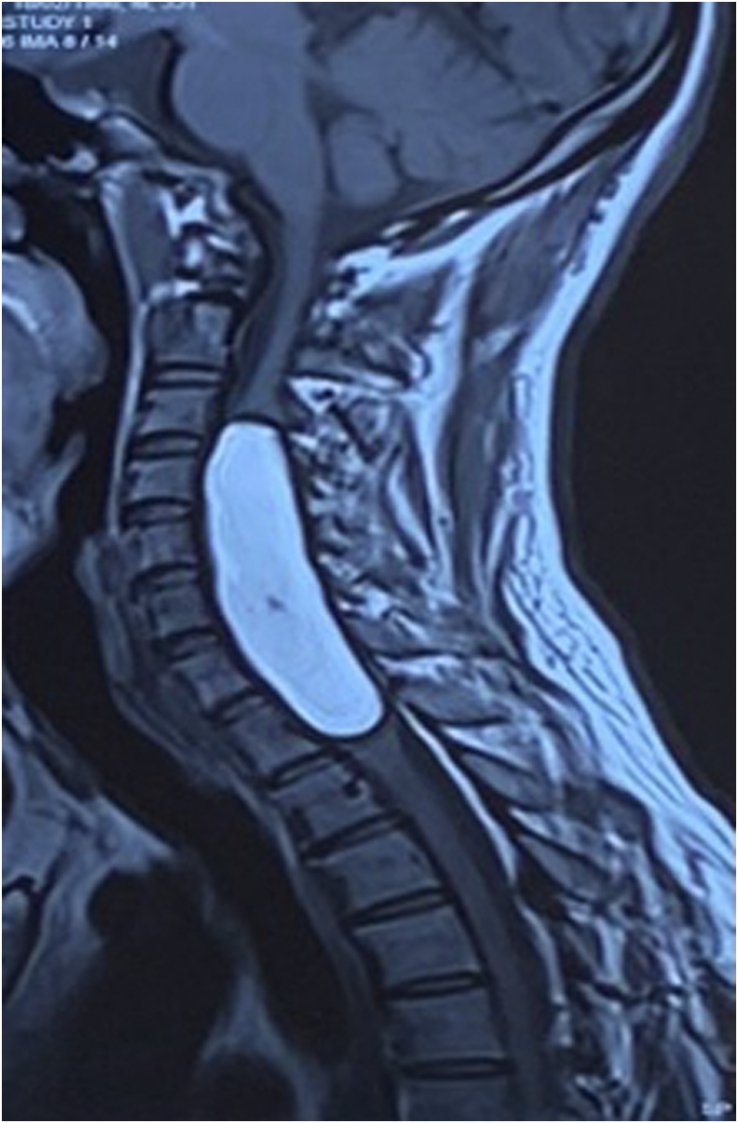
T1 weighted sagittal image showing high signal intensity lesion in cervical dorsal spine.

**Figure 3:**
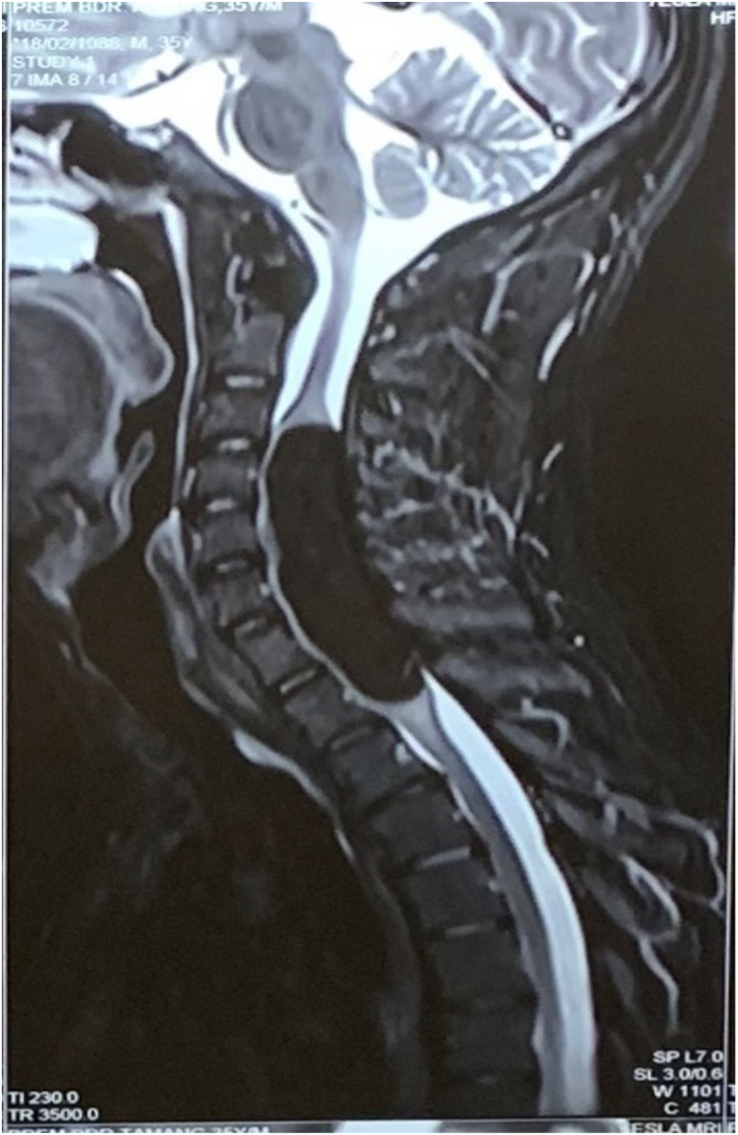
T2 STIR fat suppressed image of cervicodorsal spine shows loss of T2 high signal intensity of the lesion seen on T2W weighted sequence, suggestive of fatty nature of the lesion.

**Figure 4:**
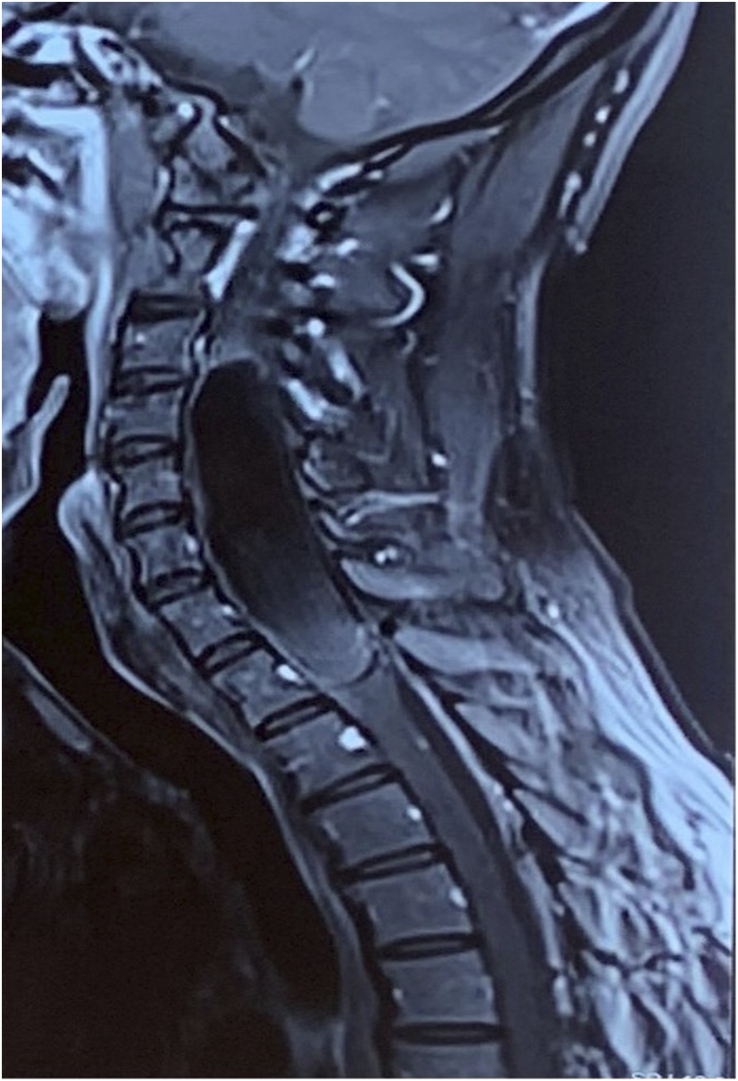
T1 weighted post gadolinium image shows no significant enhancement with thin and smooth rim enhancement.

**Figure 5:**
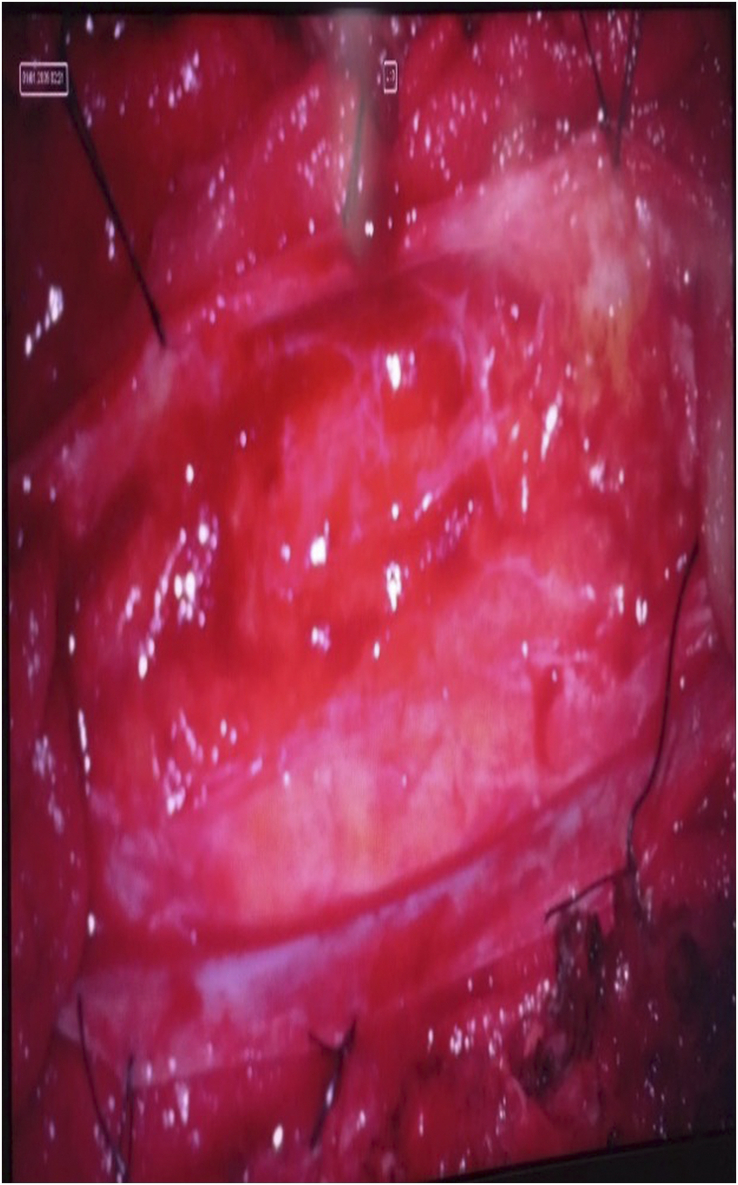
Intraoperative picture showing intramedullary lipoma.

**Figure 6:**
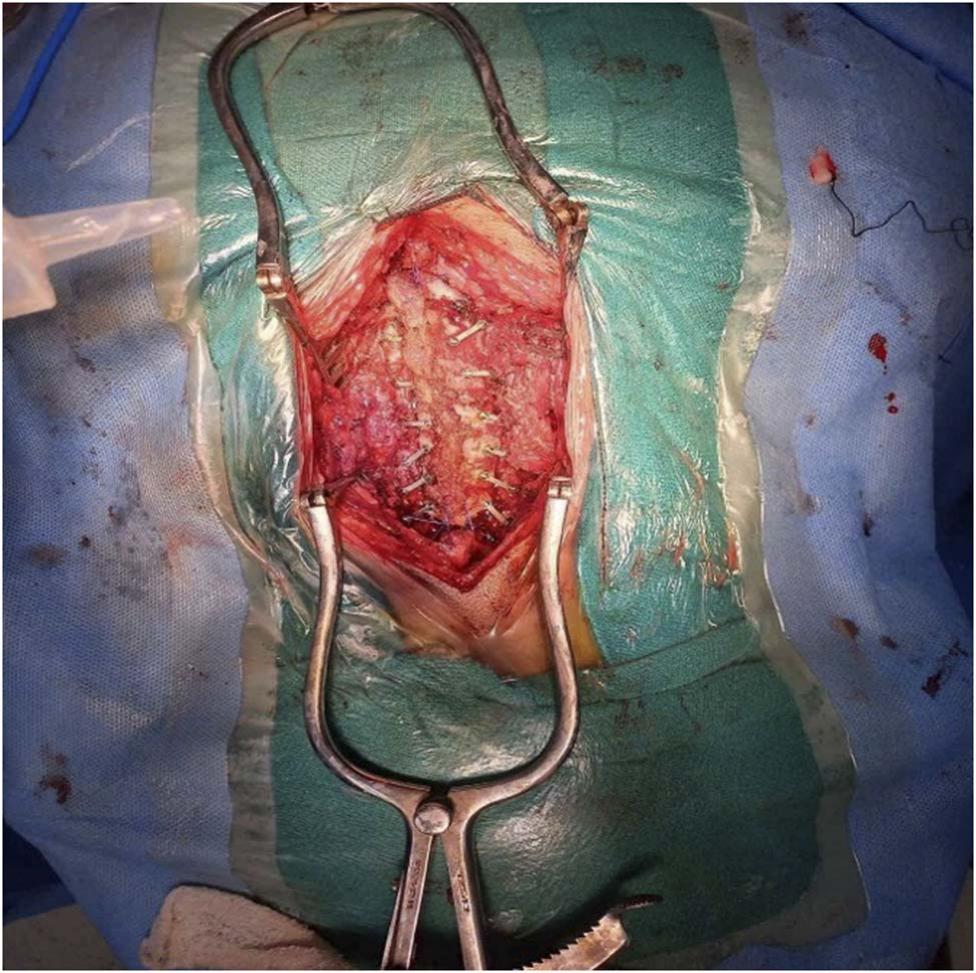
Intraoperative picture after debulking of the tumor.

**Figure 7:**
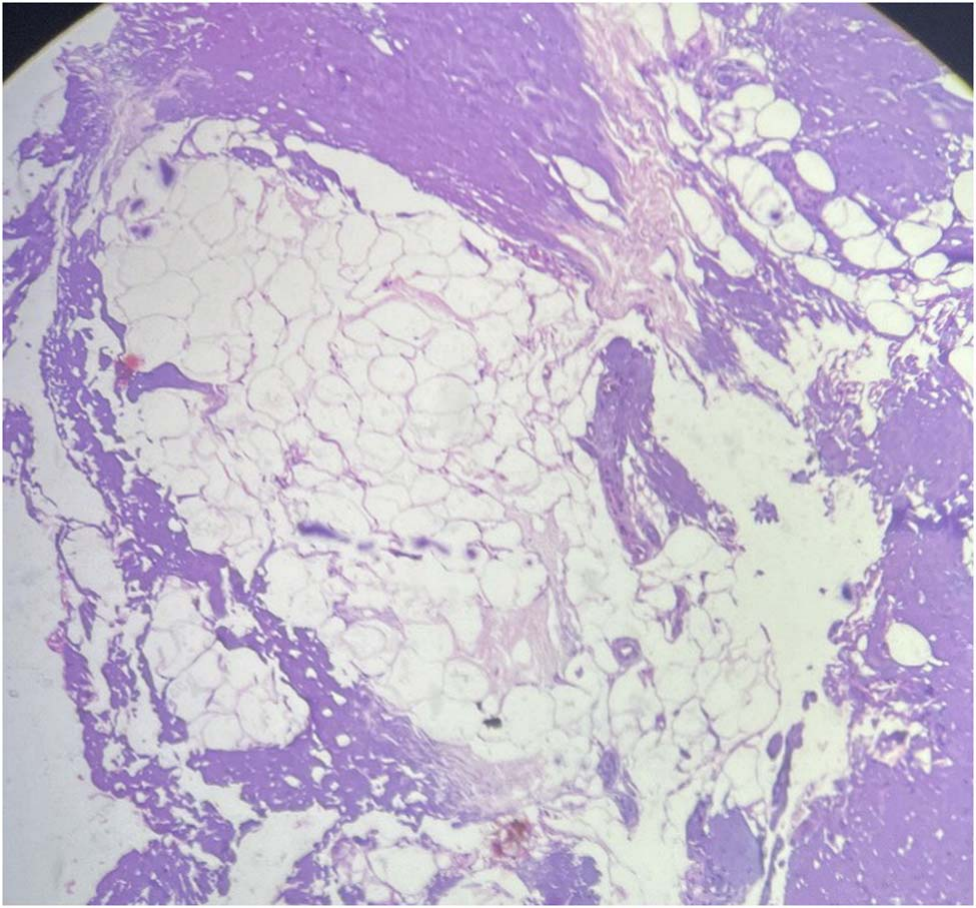
Histopathological examination showed an encapsulated tissue composed of lobules of mature adipose tissues separated by the fibrovascular septa.

The routine blood investigation, including the metabolic panel and vitamin levels, was sent along with spine computed tomography and MRI. The computed tomography showed no vertebral anomaly, and the blood investigation reports were within normal limits, ruling out most of our differentials. The MRI spine showed hyperintensities on T1 and T2 images and hypo intensity on fat suppression, suggesting cervicothoracic intramedullary lipoma extending from C3 to T1, measuring 7.6×1.8×1.5 cm without features of spinal dysraphism (Figs 1, 2, 3, 4). The patient was planned for neurosurgical interventions. The patient was positioned prone in MAYFIELD 3-point pin fixation. A midline posterior cervical incision over the marked point was given after confirmation by an X-ray. Then, dissection was done to expose C2 to the T2 vertebra, laminectomy was performed, and a tumor was identified. Intraoperative findings included a bulged thecal sac with an intramedullary tumor extending from C3 to T1 level, reaching the dorsal surface with fatty tissues intertwined with vessels and nerve roots with a thinned-out cord (Fig. 5). The patient underwent a C3-T1 laminotomy. A subtotal tumor excision, that is, ~70–80% resection, was done, followed by laminoplasty (Fig. 6). Intraoperative neurophysiological monitoring was done with motor evoked and somatosensory evoked potentials. D-wave corticospinal monitoring was employed. The bone was replaced with MR-compatible titanium consisting of 12 plates and 24 screws, and closure was done in layers. Finally, the dressing was done, and a cervical collar was applied. The excised mass was sent for histopathological examinations, which showed an encapsulated tissue composed of lobules of mature adipose tissues separated by the fibrovascular septa consistent with lipoma, thus confirming the diagnosis of intramedullary spinal cord lipoma (Fig. 7). The intraoperative and postoperative course was uneventful. The patient was managed in the postoperative and neurosurgery wards during the postoperative period.

Postoperatively, a repeat MRI of the cervicothoracic spine was done. A significant reduction in the size of the intramedullary lipoma was revealed, and postoperative complications were excluded. At the discharge, the Glasgow coma scale was 15/15; the pupil was 2 mm, round, regular, and reactive to light. Preoperative and postoperative neurological examinations were similar. Vitals were stable at the time of discharge. The patient was taking a normal diet and mobilizing well with support. At discharge, the patient’s medications were analgesic, gabapentin, calcium, vitamin D supplement, tizanidine, and limb physiotherapy. A repeat examination of the lower limb on follow-up revealed a normal tone and sensation with improved muscle power. Reflexes were normal bilaterally, although with the persistence of upgoing planter response. Overall, the patient had a good postoperative outcome.

## Discussion

Intramedullary lipoma is the primary tumor of the spinal cord. Primary spinal cord tumors are less frequent than their cranial counterpart and comprise 4–16% of all tumors of the central nervous system in adults. There are several hypothesis for the origin of spinal cord tumors, such as developmental error theory, metaplasia theory, and hamartomatous origin theory, among which the developmental error theory is the most accepted^5^. Intramedullary tumors are the least common form^2^. A study done by Endo *et al*.^1^ collected 1033 patients with intramedullary tumors with histopathological types mainly consisting of ependymoma followed by hemangioblastoma, astrocytoma with no patients with intramedullary lipoma, suggesting the rarity of our case. Nondysraphic intramedullary lipomas are extremely rare, comprising <1% of all intraspinal tumors, and most of the cases occur in children^6–8^.

Intramedullary lipoma has a bimodal presentation, becoming symptomatic during the first years of life or the second to fifth decade, as in our case. Lipoma, a slow-growing benign tumor, has a long-term static clinical course followed by an abrupt decline in neurological function shortly before the presentation, as seen in our case^8^. The common location of the intramedullary tumor was mainly cervical, followed by thoracic and cervical thoracic; the tumor was cervicothoracic in our case. As the clinical features of the disease are primarily defined by location and due to mass effect rather than the type of the tumor, the most common clinical presentation is sensory, such as limb paresthesia, weakness, gait disturbances, head, neck, back, and limb pain followed by bowel and bladder involvement which are seen in lesser number of the patients^1,3,8^. In our case, numbness and paresthesia of both upper and lower limbs and weakness of bilateral lower limbs were present. Thirty-seven previous reports describing 60 patients including one patient from the current case report with nondysraphic intramedullary spinal cord lipoma were found (Table 1).

**Table 1:** Summary of patients with nondysrpahic intramedullary spinal cord lipoma in the literature

Author and year	Patients (n)/sex	Age	Location	Symptoms
Kai *et al*., 2003^9^	1M	8 months	C	Quadrepareis
Iwatsuki *et al*., 2006^10^	1F	52Y	C	Paraparesis
Moghaddam *et al*., 2008^11^	1F	18Y	C	Upper arm hypothesia
Chagla *et al*., 2008^12^	1M	17Y	C	Hand motor deficits, paraparesis
Khurana *et al*., 2010^12^	1M	20Y	C	Quadrepareis
Azzazi *et al*., 2010^13^	1F	50Y	C	Paraparesis
Menzilcioglu *et al*., 2014^14^	1M	52Y	C	Upper arm hypothesia
Son *et al*., 2014^15^	1M	49Y	C	Left hand numbness and parapresis
Farschtschi *et al*., 2015^16^	1M	4Y	C	Hemiparesis
Severino *et al*., 2017^17^	1M	39Y	C	Quadreparesis
Kouki *et al*., 2017^18^	1M	40Y	Holocord	Not reported
Carrasco *et al*., 2018^19^	1M	47Y	C	Paraparesis
Panagopoulos *et al*., 2018^20^	1F	12Y	C	Quadreparesis
Lui *et al*., 2021^21^	1M	32Y	C	Quadreparesis
Poggi *et al*., 2021^22^	2 (1F,1M)	56Y, 11Y	CT,C	Quadreparesis, upper limb hypoesthesia
Grasso *et al*., 2023^23^	1M	32Y	C	Quadreparesis
Balachandar *et al*., 2022^24^	1F	17Y	T	Progresssive b/l lower limb weakness
Rizzuto *et al*., 2023^25^	1M	64Y	T	Bilateral lower limb myelopathy resulting in falls and difficult ambulating
Sagenly *et al*., 2023^26^	3 (2M,1F)	5 months, 2Y, 10Y	C, T, T	Progressive hypotonia of upper limb, right sided hemiparesis, backstiffness and back pain
Meher *et al*., 2017^27^	1M	30Y	C	Weakness of left shoulder and forearm
Kouadria *et al*., 2018^28^	1M	9Y	CT	Flaccid quadriplegia
Ahmed *et al*., 2015^29^	1F	31Y	C	Numbness and burning sensation on bilateral forearm
Bhatoe *et al*., 2005^3^	14 (10M, 4F)	43Y, 32Y, 37Y, 40Y, 12Y, 42Y, 24Y, 31Y, 23Y, 30Y, 28Y, 30Y, 6Y, 27Y	C,T,CT,CT,L,L,CT,CT,CT,CT,C, T, CT, C, CT	Quadriplegia/ paraparesis
Nguyen *et al*., 2017^30^	1M	2 month	CTL	Right leg plegia
Lee *et al*., 2023^8^	2F	6 months, 2 months	CT, C and T and L	Weakness of lower extremity, quadriparesis
Roohollahi *et al*., 2023^31^	1F	56Y	C	Clumsiness of right upper and lower limb
Fleming *et al*., 2010^32^	5 (2M, 3F)	2 months, 13 months, 2Y 9 months, 2Y 11 months, 4Y 9 months	CT	Asymptomatic, weakness of lower limb, pain
Kumar *et al*., 2011^33^	1M	14 months	Holodorsal	Weakness of lower limbs
Morioka *et al*., 2021^33^	1M	6Y	L	Myoclonus of lower limbs
Ali *et al*., 2022^34^	1F	21Y	T	Backache, weakness of lower limbs
Srinivasan *et al*., 2014^6^	3M	25Y, 26 Y, 25 Y	L,T, T	Low backache, dysesthesia, urinary incontinence
Yilmaz *et al*., 2018^35^	1F	3Y	T	Backache and difficulty in walking
Raj *et al*., 2023^36^	1F	9Y	T	Progressive paraplegia
Iplikcioglu *et al*., 2018^37^	1M	28Y	C	Numbness and paresthesia of upper limb and neck pain
Muthusubramanian *et al*., 2008^38^	1F	16Y	C and L	Neck pain and low badck pain with radiation
Mostarchid *et al*., 2001^39^	1M	26Y	CT	Upper thoracic pain and bilateral dysesthetic leg pain
Current case	1M	35Y	CT	Numbness and tingling sensation over bilateral upper and lower limbs

C, cervical; CT, cervicothoracic; F, female; L, lumbar; M, male; T, thoracic; Y, year(s).

MRI is the most sensitive imaging protocol; typical radiologic appearances of hyperintensity signals in T1 and T2 weighted images and demonstrating fat suppression images can confirm the diagnosis and even avoid biopsy^6,40^. Our case also showed a similar finding suggestive of intramedullary lipoma extending from C3 to T1, and we planned our patient for decompression. Gross total, subtotal, or partial resection is practiced depending on the scenario. For better postoperative neurologic outcomes, early surgical intervention and partial or subtotal resection can be performed^1,3,8,41^. Intramedullary lipoma has a benign nature, and there is a high probability of neurological deterioration due to dense adhesion between lipoma and neural tissue, as seen in our case; hence, subtotal tumor excision, that is, ~70–80% resection was done^3^. Intraoperative neurophysiological monitoring was done with motor evoked and somatosensory evoked potentials. D-wave corticospinal monitoring was employed. Neuromonitoring helps prevent spinal injury, improving postoperative neurologic functioning and outcome^42^. The role of intraoperative ultrasonography is important as it helps delineate the extent of lipoma due to its hypoechoic nature^43^. Intramedullary lipoma is a slow-growing tumor; recurrence is inevitable. There is no difference between recurrence in the resection modalities, either gross total, subtotal, or partial resection^6^. There are different proposed theories for its pathogenesis, such as malformation or neoplastic, metabolic, and hormonal, but clear etiology is still lacking. The study done by Endoh *et al*.^44^, Akyuz *et al*.^45^, and Fleming *et al*.^32^ aligns with the hormonal and metabolic theory and claims to have recurrence more among the young population. Neuroimaging and clinical symptoms can identify recurrence, and if it recurs, re-debulking at an earlier stage helps preserve neurological function^6^.

## Conclusion

Intramedullary nondysraphic spinal lipomas are extremely rare among primary spinal cord tumors. These patients present with nonspecific sensory symptoms followed by deterioration of motor symptoms. As the safety margins for neurological preservation are thin, meticulously locating the extent of the tumor, and choosing the resection modalities is essential. We report a case of a 35-year-old male diagnosed with nondysraphic intramedullary cervicothoracic lipoma who underwent subtotal resection of the tumor.

## Ethical approval

Approval is not required at our institution for the publication of the individual case.

## Consent

Written informed consent was obtained from the patient for publication and any accompanying images. A copy of the written consent is available for review by the Editor-in-Chief of this journal on request.

## Sources of funding

Not applicable.

## Author contribution

Dr S.K., Dr P.R., Dr A.P., and Dr K.K.: conceptualization, data collection, drafting of the original manuscript, and revision of the manuscript; Dr B.T.: data collection, drafting the original manuscript, and revision of the manuscript; Dr S.K.S.: in charge of the case and did the final revision of the manuscript.

## Conflicts of interest disclosure

The authors declare no conflicts of interest.

## Research registration unique identifying number (UIN)

Our institution does not require IRB clearance for the case reports.

## Guarantor

Dr Susmin Karki.

## Data availability statement

A copy of the written consent is available for review by the Editor-in-Chief of this journal on request.

## Provenance and peer review

Not commissioned, externally peer-reviewed.
